# Integrative Identification of Anti‐Photoaging Peptides From Stress‐Tolerant Microorganisms via Machine Learning and KEAP1–NRF2 Docking

**DOI:** 10.1002/psc.70115

**Published:** 2026-07-01

**Authors:** Hanui Lee, Gyeong Han Jeong, Ji Wan Choi, Taehwan Kim, Younhee Shin, Jaewon Lim, Kwang‐Woo Jung, Byung Yeoup Chung, Seung Sik Lee

**Affiliations:** ^1^ Research Division for Biotechnology, Advanced Radiation Technology Institute (ARTI) Korea Atomic Energy Research Institute (KAERI) Jeongeup Republic of Korea; ^2^ Department of Radiation Science University of Science and Technology (UST) Daejeon Republic of Korea; ^3^ Research and Development Center Insilicogen Inc Yongin‐si Gyeonggi‐do Republic of Korea

**Keywords:** antioxidant peptides, KEAP1–NRF2, machine learning, molecular docking, photoaging

## Abstract

Excess reactive oxygen species generated by ultraviolet exposure cause photoaging by degrading collagen and inhibiting its synthesis. This study presents a comprehensive strategy connecting the biological stress responses of γ‐irradiated microorganisms to the discovery of novel anti‐photoaging peptides. We profiled the radiation‐regulated transcriptomes of *Deinococcus radiodurans* and *Cryptococcus neoformans* , focusing on DNA repair and oxidative stress responses. From these datasets, peptide libraries were generated in silico, filtered for biochemical properties, and prioritized using a seven‐classifier machine‐learning algorithm. Structural validity was established using Rosetta FlexPepDocking against the KEAP1–NRF2 pocket, which identified 48 docking‐positive sequences. We then synthesized the top 21 peptides and subjected them to in vitro validation. Seven of these candidate peptides inhibited collagenase activity at 200 μM. Among them, four peptides dose‐dependently increased the procollagen type I C‐peptide level in ultraviolet B‐induced fibroblasts. Furthermore, these peptides significantly elevated COL1A1 mRNA levels while simultaneously reducing MMP1 and MMP9 transcript and protein levels. In summary, this study provides an integrated strategy that combines omics, machine learning, and docking to discover promising peptide candidates, which were validated through in vitro assessments. This approach offers promising anti‐photoaging candidates that can be applied to other oxidative stress pathways and biological resources.

## Graphical abstract

Novel short‐chain peptides targeting the KEAP1‐NRF2 axis were identified through a multimodal discovery platform combining a stress‐tolerant microbial library with machine learning and molecular docking. These peptides regulate collagen synthesis and reduce MMP1/9 levels in human fibroblasts, providing significant protection against photoaging.

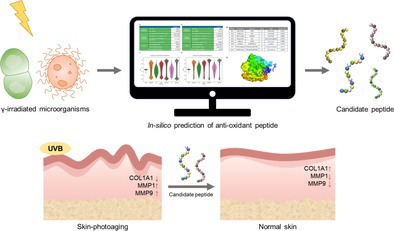

## 1 Introduction

Skin aging, particularly photoaging, is primarily caused by reactive oxygen species (ROS) accumulation resulting from chronic exposure to ultraviolet (UV) radiation. Excess ROS levels cause oxidative stress, which accelerates inflammatory signaling, induces cell damage, promotes pigmentation, and contributes to aging [1, 2]. In UV‐exposed skin, matrix metalloproteinase (MMP) expression increases, whereas type I collagen synthesis decreases, resulting in structural integrity loss and wrinkle formation [3, 4]. Therefore, strategies that inhibit collagen degradation and restore collagen synthesis are crucial for anti‐photoaging effects.

Short peptides are attractive candidates for various applications owing to their favorable safety profile, adjustable physicochemical properties, unique targeting potential, and flexibility in formulation [5, 6, 7]. Although various studies have focused on food source‐ or organism‐derived bioactive peptides, most lack clearly defined mechanisms of action [8]. In particular, the rational design of peptides that utilize endogenous antioxidant signaling, such as Kelch‐like ECH‐associated protein 1 (KEAP1)–nuclear factor erythroid 2‐related factor 2 (NRF2) complexes, remains unexplored in the discovery of anti‐photoaging peptides.

The KEAP1–NRF2 pathway is a crucial mechanism that regulates cellular responses to oxidative stress [9]. Under stressful conditions, Nrf2 dissociates from Keap1 and translocates to the nucleus, triggering antioxidant gene expression [10]. Therefore, peptides inhibiting the binding between Keap1 and Nrf2 or promoting Nrf2 activation could help repair oxidative stress‐mediated cellular damage. Microorganisms that adapt to extreme environments possess a unique stress tolerance, particularly in response to radiation and oxidative damage [11, 12]. Proteins and peptides are valuable sources of antioxidant candidates. Notably, *Deinococcus radiodurans* is well known for its resistance to radiation, whereas *Cryptococcus neoformans* is recognized for its adaptability to various environmental stresses [13, 14]. Therefore, a well‐considered strategy targeting the stress‐adapted proteomes of these microorganisms is essential.

In this study, we developed an integrated workflow that links the biological stress responses of γ‐irradiated *D. radiodurans* and *C. neoformans* to the identification of potential anti‐photoaging peptide candidates. We first performed RNA sequencing (RNA‐seq) to profile the transcriptome data and define radiation‐responsive signatures. These proteins were then used as sources for in silico peptide generation. Next, we prioritized candidate peptides by applying sequential biochemical property filtering followed by machine learning (ML) screening using seven classifiers. To establish the structural basis for our findings, we conducted molecular docking of the KEAP1–NRF2 pocket. The top‐ranked short peptides were subjected to functional assays. To validate the anti‐photoaging efficacy of the selected candidate peptides, we performed collagenase activity assays and in vitro analyses to measure procollagen type I C‐peptide (PIP), MMP1, and MMP9 expression levels in UVB‐exposed human fibroblasts.

This study aimed to systematically explore stress‐adapted proteomes derived from γ‐irradiated microorganisms to identify antioxidant peptides targeting the KEAP1–NRF2 axis and validate their anti‐photoaging activity at the cellular level. This approach will enhance the rational design of functional peptides from unique microbial resources, thereby expanding their potential as antioxidant and anti‐photoaging agents. Taken together, this strategy, which combines omics, ML, and docking, was developed to identify short peptides with anti‐photoaging properties. Our findings highlight the specific peptides that suppress collagenase activity and promote procollagen biosynthesis in UVB‐stimulated fibroblasts. These findings highlight their potential as anti‐photoaging agents and demonstrate a broad strategy for the discovery of functional peptides.

## 2 Materials and Methods

### 2.1 Cell Strains and Gamma Irradiation

*C. neoformans* strains were cultured in YPD medium (1% yeast extract, 2% peptone, 2% dextrose) at 30°C with shaking. *D. radiodurans* R1 (ATCC 13939) was grown in TGY broth (0.5% tryptone, 0.1% glucose, 0.3% yeast extract) at 30°C with shaking. Overnight stationary‐phase seed cultures were diluted 1:100 in fresh medium for the experiments. *C. neoformans* and *D. radiodurans* were irradiated with 2 and 10 kGy UVB, respectively, at the Advanced Radiation Technology Institute, Korea Atomic Energy Research Institute (Jeongeup, Korea) using a cobalt‐60 irradiator (point source AELC; IR‐79; MDS Nordion International Co. Ltd.) with approximately 320 kCi source strength and 10 kGy/h dose rate.

### 2.2 Total RNA Isolation and RNA‐seq

Total RNA was extracted from the irradiated cells using TRIzol reagent (EasyBlue) according to the manufacturer's protocol [15]. The RNA quantity and purity were assessed using a NanoDrop Lite spectrophotometer (Thermo Fisher Scientific) at an absorbance of A260/A280. The QC‐passed samples were sequenced on an Illumina NovaSeq 6000 paired‐end sequencing platform. Sequencing adapters were trimmed using Trimmomatic v0.39, and the reads were aligned against reference genomes ( *C. neoformans* var. *grubiiH99*, *D. radiodurans* *R1*) using STAR v2.7.11b. Gene‐level counts were estimated using RSEM v1.3.1 and differentially expressed genes (DEGs) between samples were identified using edgeR v3.30.3.

### 2.3 In Silico Peptide Preparation and Physicochemical Property Screening

Peptides from the protein sequences were generated using the Epestfind and rapid peptide generator (RPG) programs. Bioactive peptides were predicted based on their physicochemical properties. In brief, physicochemical properties were analyzed using EMBOSS Pepstats, and in vitro and in vivo aggregation was determined using Tango and Aggrescan tools, respectively. The thresholds used for filtering are listed in Table 1.

**TABLE 1:** Biochemical properties prediction and filtration of peptides.

Parameters	Tools	Cutoff	# of sequences	
			*C. neoformans*	*D. radiodurans*
Isoelectric point	PEPSTATS	8 ≤ pI ≤ 12	2,466,490	556,280
Length	PEPSTATS	5 ≤ Length ≤ 9	3,027,636	875,088
Alpha helix propensity	TANGO	0 ≤ HELIX ≤ 25	7,425,659	1,931,289
Beta sheet propensity	TANGO	25 ≤ BETA ≤ 100	2,806,983	674,803
In vitro aggregation	TANGO	AGG ≤ 500	7,558,498	1,977,691
In vivo aggregation	AGGRESCAN	−40 ≤ Na^4^vSS ≤ 60	5,329,085	1,498,693
Final			516,357	138,210

### 2.4 Novel Antioxidant Peptide Selection Using ML

To identify the novel antioxidant peptides, the models were trained using antioxidant prediction models. The positive dataset, consisting of 621 unique experimentally validated antioxidant peptides, was comprehensively collected from six major public repositories (Antioxidant Database [AODB], Antioxidant Protein Database [AOD], AnOxPePred, AutoPeptideML, BIOPEP‐UWM, and Database of Food‐Derived Bioactive Peptides [DFBP]). To eliminate potential dataset bias and perform rigorous binary classification, a positive dataset consisting of 621 non‐antioxidant peptides was generated using AnOxPePred and Peptipedia. Initially, 73 verified non‐antioxidant peptides were derived from AnOxPePred. To supplement the remaining negative data, 1000 candidate sequences without antioxidant labels were randomly selected from Peptipedia, followed by stringent sequence identity filtering using CD‐HIT (threshold < 0.5) to yield 548 structurally distinct negative peptides. Peptide sequences were converted into numerical feature vectors using the MathFeature package. The extracted feature representations included AAC, AAindex, ComplexNetwork, DPC, Kgap, MappingProtein, TPC, Tsallis, and TsallisEntrop. The detailed types and numbers of these features are listed in Table 2. In total, 12,648 unique features were generated and used as input for ML models. The ML models used in this study were the Ada Boost Classifier (ada), CatBoost Classifier (catboost), Extra Trees Classifier (et), Gradient Boosting Classifier (gbc), Light Gradient Boosting Machine (lightgbm), Randon Forest Classifier (rf), and Extreme Gradient Boosting (xgboost). The trained models were then tested and evaluated with the testing (559 positive and 559 negative) and evaluation sets (62 positive and 62 negative). A peptide was considered an antioxidant candidate if at least five models predicted a positive label.

**TABLE 2:** Types and numbers of feature representations used in this study.

Feature ID	AAC	AAindex	ComplexNetwork	DPC	Kgap	MappingProtein	TPC	Tsallis	TsallisEntrop	Total
Feature number	20	3472	240	400	400	110	8000	3	3	12,648

### 2.5 Molecular Docking Analysis

Molecular docking against the KEAP1–NRF2 domain was performed using Rosetta FlexPepDock to predict the antioxidant candidate peptides. The 3D structure of Keap1 used for docking was recovered from the protein data bank (PDB: 2FLU). The receptor and peptide ligands were prepared by removing heteroatoms and water, adding hydrogen atoms, and defining the binding cavity based on a known NRF2‐binding site. For each peptide, we performed 10 independent docking experiments and used the average score to rank the peptides. Candidate peptide interactions with the targets were observed and analyzed using PyMOL. The control for molecular docking comprised NRF2‐derived ETGE/DLG motifs and test peptides (glutathione, peptuna, synthetic, NRF2modelled, and NRF2Original). Peptides with an average score ≤ −8.07 (based on the NRF2 modeled) were considered docking positive. Among these, the top 12 short peptides from *C. neoformans* and the top 9 short peptides from *D. radiodurans* were selected and chemically synthesized by GenScript (Nanjing, China) with a purity of > 95%. The chemical identity and purity of the peptides were further confirmed by high‐performance liquid chromatography (HPLC) and mass spectrometry (MS) analysis.

### 2.6 Collagenase Inhibition Assay

To assess the inhibitory effects of the candidate peptides, collagenase activity was measured using a Collagenase Activity Assay Kit (Abcam) according to the manufacturer's instructions. The synthetic peptide FALGPA was used as the substrate and 1, 10‐phenanthroline was used as an inhibitor. Peptide samples, positive controls, and inhibitors were mixed with collagenase assay buffer and substrate. The absorbance of the mixtures was immediately measured at 345 nm on a microplate reader in kinetic mode for at least 15 min at 37°C. The collagenase inhibition activity in the sample was calculated as follows: [(activity of collagenase control − activity of sample control) / activity of collagenase control] × 100.

### 2.7 Cell Culture and Cell Viability Assay

Human fibroblast cell line CCD‐986sk was purchased from the Korean Cell Line Bank (Seoul, Korea) and cultured in Iscove's Modified Dulbecco's medium (IMDM) supplemented with 10% FBS and 1% (v/v) penicillin–streptomycin in a 37°C humidified incubator with 5% CO_2_. The effect of the candidate peptides on cell viability was assessed using the conventional 3‐(4,5‐dimethylthiazol‐2‐yl)‐2,5‐diphenyltetrazolium bromide (MTT) assay [16]. Briefly, 5 × 10^4^ cells were seeded in a 96‐well plate and cultured for 24 h at 37°C. After removing the culture medium, the cells were treated with the candidate peptides and incubated for 24 h. A 0.5 mg/mL MTT solution was added to each well and incubated for 3 h. After 3 h, the formazan crystals formed were dissolved in 100 μL DMSO. Absorbance was measured using a microplate reader (Infinite F200; Tecan, Austria) at 570 nm. Cell viability based on the measured optical density (OD) was determined by comparing the absorbance with that of control cells.

### 2.8 Measurement of Procollagen and Metalloproteinases Expression

To induce photoaging in CCD986‐sk cells, the cells were exposed to 50 mJ/cm^2^ UVB radiation with a mid‐range wavelength of 302 nm using a UVP Crosslinker CL‐3000 M (Analytik Jena, Germany). After UVB exposure, the cells were treated with 50, 100, and 200 μM peptide and incubated at 37°C for 72 h. PIP EIA Kit (Takara, Korea), MMP‐1 Human ELISA Kit, and MMP‐9 Human ELISA Kit (Invitrogen, USA) were used to detect the protein concentrations according to the manufacturer's instructions.

### 2.9 Quantitative PCR (qPCR)

Total RNA from peptide‐treated CCD986‐sk cells was extracted using the WelPrep Total RNA Isolation Reagent (Welgene) and cDNA was synthesized using a PrimeScript II 1st Strand cDNA Synthesis Kit (Takara, China). After cDNA synthesis, quantitative reverse transcription polymerase chain reaction (qRT‐PCR) was performed using SYBR PCR Master Mix (Bio‐Rad, USA), according to the manufacturer's protocol. *β‐actin* was used as the housekeeping gene. Gene expression changes in the samples were expressed as fold changes relative to the control group. The statistical analysis was performed using the 2^−ΔΔCt^ method. The primer sequences used in this study were as follows: *Col1A1* (forward: primer 5′‐GGAGAGAGCATGACCGATGG‐3′; reverse: 5′‐CGATCTCGTTGGATCCCTGG‐3′), *MMP‐1* (forward: 5′‐ACAGGCTCCGAGAAATGCAA‐3′; reverse: 5′‐TTCACCCACATCAGGCACTC‐3′), *MMP‐9* (forward: 5′‐CAGACCAAGGGTACAGCCTG‐3′; reverse: 5′‐ATACAGCGGGTACATGAGCG‐3′), and *β‐actin* (forward: 5′‐CATGAAGTGTGACGTGGACA‐3′; reverse: 5′‐CAGGGCAGTGATCTCCTTCT‐3′).

### 2.10 Statistical Analysis

All statistical analyses were performed using the GraphPad Prism software (GraphPad Software, USA). Student's *t*‐test was used to compare groups. Data are expressed as the mean ± standard deviation (SD). The *p*‐values in the multiple comparison results (*, **, and ***) indicate significant differences between groups (*p* < 0.05, *p* < 0.01, and *p* < 0.001, respectively).

## 3 Results

### 3.1 Transcriptomic Profiling of Irradiated *D. radiodurans* and *C. neoformans*

**FIGURE 1:**
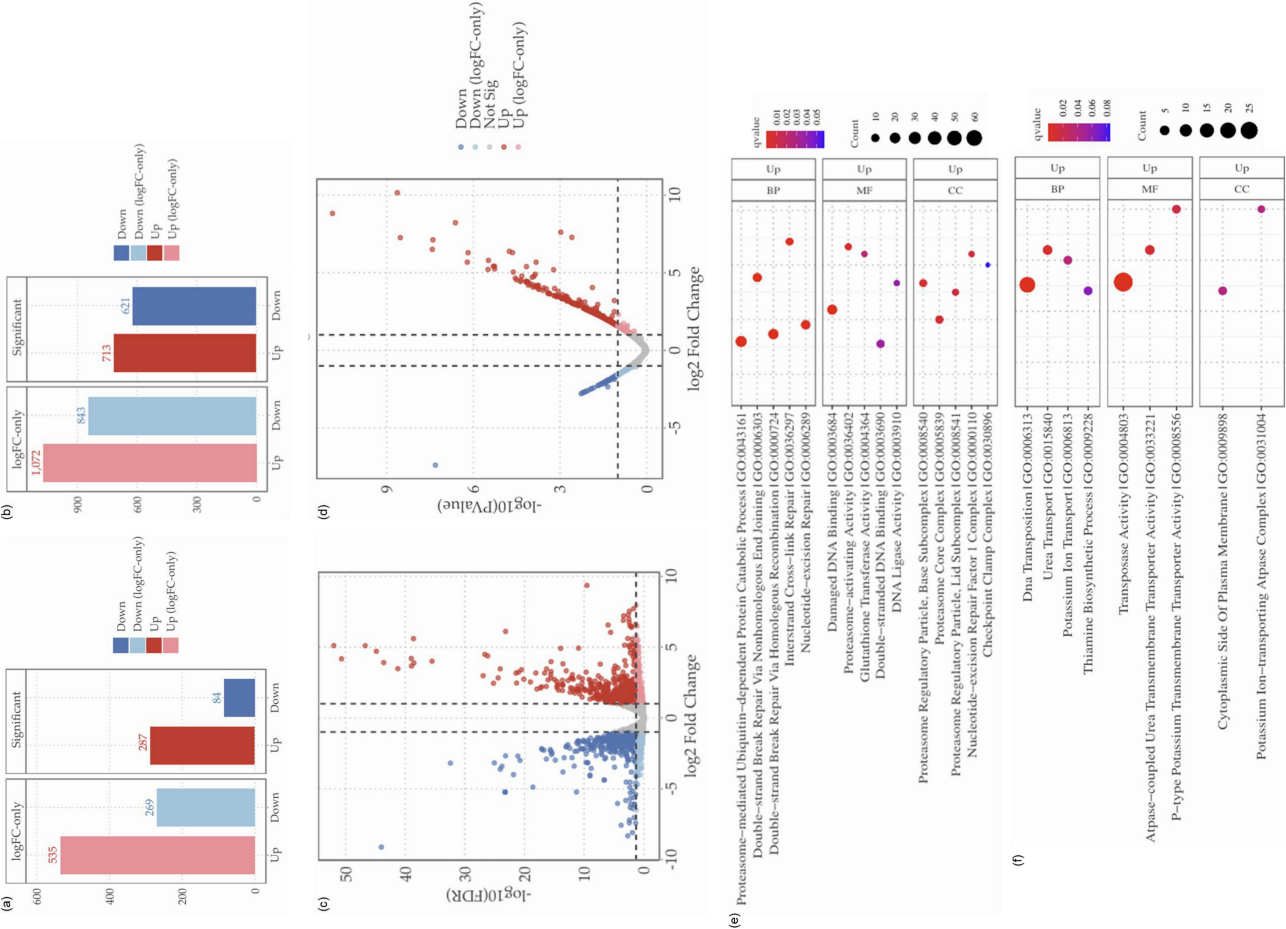
Transcriptomic responses to γ‐irradiation. (a) Number of differentially expressed genes (DEGs) in *C. neoformans* and *D. radiodurans* (irradiated vs. basal), partitioned into up‐ and down‐regulated sets. (b, c) Volcano plots showing log2 fold changes and statistical significance for *C. neoformans* (b) and *D. radiodurans* (c). (d) GO enrichment summaries of irradiation‐responsive pathways in each species, highlighting DNA damage repair, ubiquitin–proteasome processes, antioxidant detoxification, and ion/urea transport.

To explore the regulatory effects of γ‐irradiation on *D. radiodurans* and *C. neoformans* , we performed RNA‐seq. The total read bases and GC contents are summarized in Table 3. We profiled the gene‐level expression abundance and differential patterns between irradiated and non‐irradiated groups. Differential expression analysis identified 1334 and 371 DEGs in *C. neoformans* and *D. radiodurans* , respectively. Among these, 718 and 287 genes were upregulated in *C. neoformans* and *D. radiodurans* , respectively (Figure 1a–d). To further explore the biological pathways associated with these DEGs, we performed GO enrichment analyses. Notably, functional enrichment analyses in both organisms revealed shared mechanisms consistent with radiation‐induced damage repair and mitigation. Specifically, the enrichment analysis showed significant upregulation of pathways related to DNA damage repair, proteasome‐mediated repair of degraded proteins, lipid transport, and ATPase activity in *C. neoformans* (Figure 1e). Similarly, irradiation upregulated the DNA transposition, potassium ion transport, energy metabolism, and cytoplasmic function regulation pathways in *D. radiodurans* (Figure 1f). These similarities in transcriptomic profiles implicate the potential antioxidant activities of peptides derived from γ‐irradiated microorganisms, providing a biological rationale for the subsequent functional validation of peptides targeting the KEAP1–NRF2 pathway.

**TABLE 3:** Summary of sequencing data.

Sample	Total bases (bp)	Total reads	GC content (%)	Q20 (%)	Q30 (%)
Crypto_basal	7,233,155,592	71,516,392	52.1	99.0	96.8
Crypto_radiation	7,550,374,180	74,555,540	49.8	98.9	96.6
Deino_basal	5,702,025,296	56,455,696	68.0	98.2	95.0
Deino_radiation	5,845,741,428	57,878,628	67.5	98.2	95.2

*Note:* Clean reads were paired‐end reads of clean data.

### 3.2 ML‐Based Prioritization of Antioxidant‐Peptide Candidates

**FIGURE 2:**
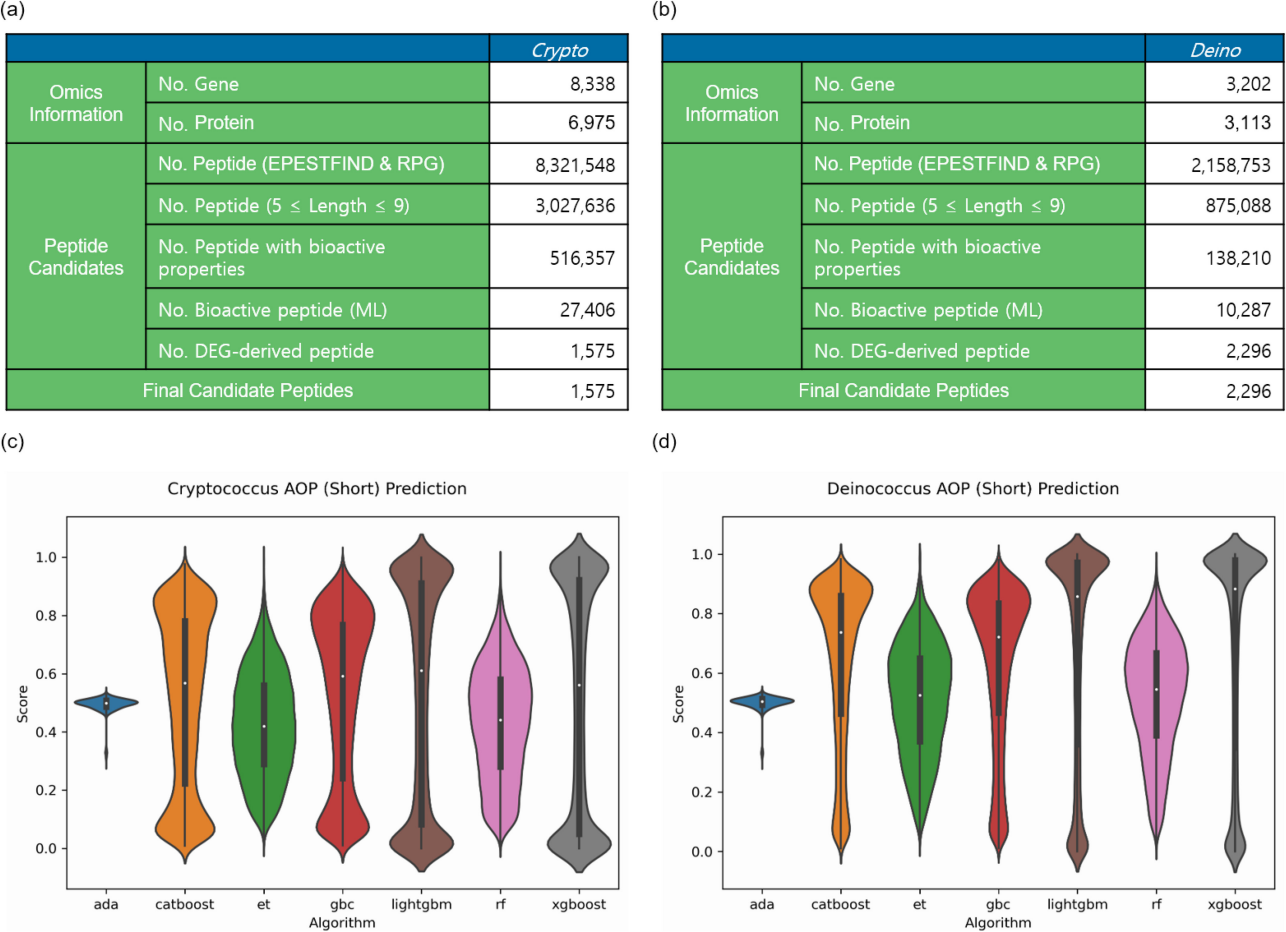
Machine learning (ML) prioritization of antioxidant‐peptide candidates. (a) Stepwise screening counts after in silico digestion and filtering: ML consensus (positive in ≥ 5/7 classifiers) retained 38,451 sequences in *C. neoformans* and 46,809 in *D. radiodurans* . (b, c) Violin plots of predicted antioxidant scores (0–1) across seven classifiers for short peptides from *C. neoformans* (b) and *D. radiodurans* (c). White dot, median; gray box, IQR. Dashed lines denote model‐specific hit thresholds (ADA = 0.53; others = 0.80). Peptides exceeding thresholds in ≥ 5/7 models were advanced to docking.

ML is a powerful method for predicting antioxidant potential by identifying preliminary patterns in large‐scale datasets [17]. In this study, we employed seven classifiers to prioritize the peptides derived from γ‐irradiated microorganisms before docking to the KEAP1–NRF2 binding site. Putative peptides were generated from gamma‐irradiated proteins. Then, a two‐step screening process involving biochemical properties (pI, secondary structure, and in vitro/in vivo aggregation) and ML training was applied to prioritize sequences with antioxidant potential (Figure 2). Figure 2a shows the stepwise screening yields for *C. neoformans* and *D. radiodurans* . After *in silico* digestion and sequential filtering based on length and biochemical properties, the ML model prioritized 38,451 and 46,809 peptides from *C. neoformans* and *D. radiodurans* , respectively. The violin plot distribution of *C. neoformans* peptide samples from the seven independently trained models is shown in Figure 2b, and a similar pattern was observed for *D. radiodurans* (Figure 2c). The ML‐ranked peptides were selected for further docking analyses.

### 3.3 In Silico Molecular Docking of the Candidate Peptides

**FIGURE 3:**
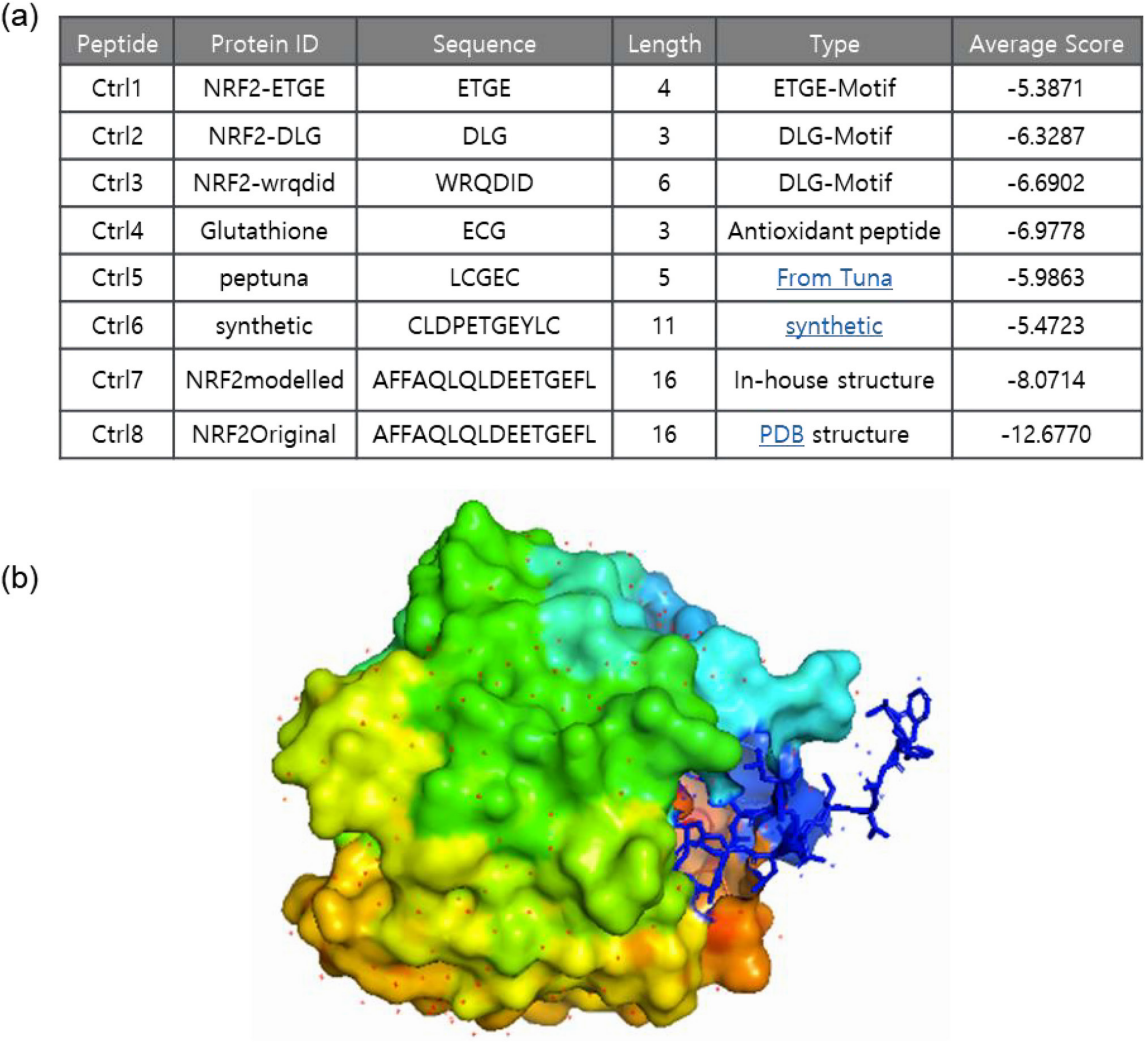
Docking control calibration at the KEAP1 NRF2 site. (a) Mean docking scores for reference controls at the KEAP1 NRF2‐binding pocket (PDB 2FLU): NRF2‐Original, NRF2‐modeled, ETGE, DLG, and glutathione; 50 replicate runs per control. Thresholds used in this study were −8.07 (NRF2‐modeled). (b) Control structure of the KEAP1–NRF2 complex (PDB: 2FLU) highlighting the NRF2‐recognition pocket. KEAP1 is shown as a surface; the bound NRF2 peptide (ETGE motif) is shown as blue sticks.

The KEAP1–NRF2 pathway is a key transcriptional regulator of cellular antioxidant responses. However, when oxidative stress occurs, the binding between these two molecules breaks, allowing NRF2 to translocate to the nucleus where it promotes antioxidant gene expression [18]. Therefore, peptides that release NRF2 from KEAP1 may protect against oxidative stress. Accordingly, we performed molecular docking to confirm that in silico‐prioritized peptides bind to the NRF2‐recognition pocket of KEAP1 and compared the mean scores with those of controls containing NRF2‐derived motifs and known antioxidants. Candidate peptides were docked to KEAP1 at the NRF2‐binding site using Rosetta FlexPepDock. First, the molecular docking of the controls was performed, and the average score was used for ranking. Figure 3a shows the average scores for the NRF2‐derived motif sequences and synthetic antioxidant peptides used as controls. Using the NRF2 benchmark of −8.07 derived from the NRF2 modeled control, peptides with mean docking scores < −8.07 were classified as docking positive (Figure 3b). Under this single criterion, *C. neoformans* contributed 31 peptides (best score −12.7) and *D. radiodurans* contributed 17 (−10.3) (Tables S1 and S2). Collectively, 48 short peptides were prioritized as docking‐positive candidates with predicted affinities for the KEAP1–NRF2 pocket. These results support their potential to competitively disrupt KEAP1–NRF2 interactions and motivate subsequent functional validation. Among these, the top 21 candidates with the highest docking scores were selected for chemical synthesis and subsequent functional validation. Detailed analytical data of the synthetic peptides, including HPLC chromatograms and MS spectra, are provided in Figures S1–S8.

### 3.4 Identification of Anti‐Photoaging Peptides Among In Silico‐Prioritized Candidates

**FIGURE 4:**
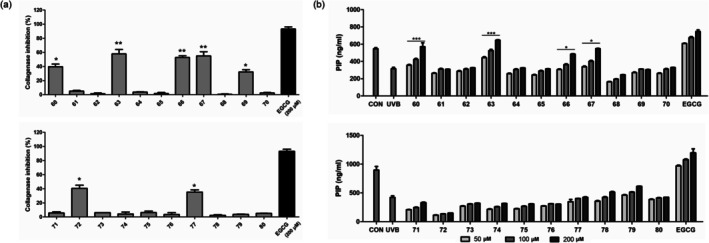
In vitro screening of candidate peptides for anti‐photoaging activity. (a) Collagenase inhibition at 200 μM for 39 candidates; 11 peptides (60, 63, 66, 67, 69, 72, 77) significantly reduced enzyme activity compared with EGCG under identical conditions. (b) Procollagen type I C‐peptide (PIP) ELISA in ultraviolet B‐challenged CCD‐986sk fibroblasts: peptides 60, 63, 66, 67 dose‐dependently restored PIP, approaching the EGCG control at 200 μM. Data are presented as mean ± SD (*n* = 3); (*p* < 0.05, *p* < 0.01, *p* < 0.001 vs. indicated controls).

To experimentally validate the 21 in silico‐predicted peptides, we synthesized and measured the collagenase inhibitory activity of the peptides and their ability to increase the expression of PIP in UVB‐induced fibroblasts. The amino acid sequences of these 21 candidates are listed in Table 4. MTT analysis was performed to determine an appropriate treatment concentration for subsequent in vitro assays. All tested candidate peptides exhibited negligible cytotoxicity against CCD‐986sk fibroblasts at 200 μM, supporting the use of this concentration for subsequent functional analyses (Figure S9). First, the anti‐collagenase activities of candidate peptides were investigated. Among the 21 candidates, only seven peptides (peptide 60, 63, 66, 67, 69, 72, 77) significantly suppressed collagenase activity at 200 μM (Figure 4a). In UVB‐exposed CCD‐986sk fibroblasts, peptides 60, 63, 66, and 67 dose‐dependently restored PIP to levels similar to those in the EGCG‐treated control (Figure 4b). Taken together, our screening data indicated that several peptides (peptides 60, 63, 66, and 67) exert a complex ECM‐protective effect, including reduced collagenase activity and procollagen restoration in UVB‐challenged fibroblasts, thereby supporting their translational relevance as anti‐photoaging agents.

**TABLE 4:** List of synthesized candidate peptides.

Peptide number	Name	Sequence
60	ISG‐P‐Crypto‐AOP‐S‐0001	VHVLIR
61	ISG‐P‐Crypto‐AOP‐S‐0588	HIAPIR
62	ISG‐P‐Crypto‐AOP‐S‐0367	IVLRPH
63	ISG‐P‐Crypto‐AOP‐S‐1036	VVRVHP
64	ISG‐P‐Crypto‐AOP‐S‐1151	MVLRVHP
65	ISG‐P‐Crypto‐AOP‐S‐1462	GTVHIR
66	ISG‐P‐Crypto‐AOP‐S‐1438	MRIHV
67	ISG‐P‐Crypto‐AOP‐S‐1069	HVITGR
68	ISG‐P‐Crypto‐AOP‐S‐1023	VIVRH
69	ISG‐P‐Crypto‐AOP‐S‐0386	HIMVRA
70	ISG‐P‐Crypto‐AOP‐S‐1082	MRIHLP
71	ISG‐P‐Crypto‐AOP‐S‐1378	NRHILP
72	ISG‐P‐Deino‐AOP‐S‐1597	HALIVRP
73	ISG‐P‐Deino‐AOP‐S‐1036	VRVPGHA
74	ISG‐P‐Deino‐AOP‐S‐0842	RVHPI
75	ISG‐P‐Deino‐AOP‐S‐1949	PAIHVGR
76	ISG‐P‐Deino‐AOP‐S‐1739	HGLPGRL
77	ISG‐P‐Deino‐AOP‐S‐0541	LHHIR
78	ISG‐P‐Deino‐AOP‐S‐0451	AHLMRP
79	ISG‐P‐Deino‐AOP‐S‐1822	LNVGRH
80	ISG‐P‐Deino‐AOP‐S‐1091	FVHVR

### 3.5 Candidate Peptides Effectively Regulate Photoaging‐Related Gene Expression

**FIGURE 5:**
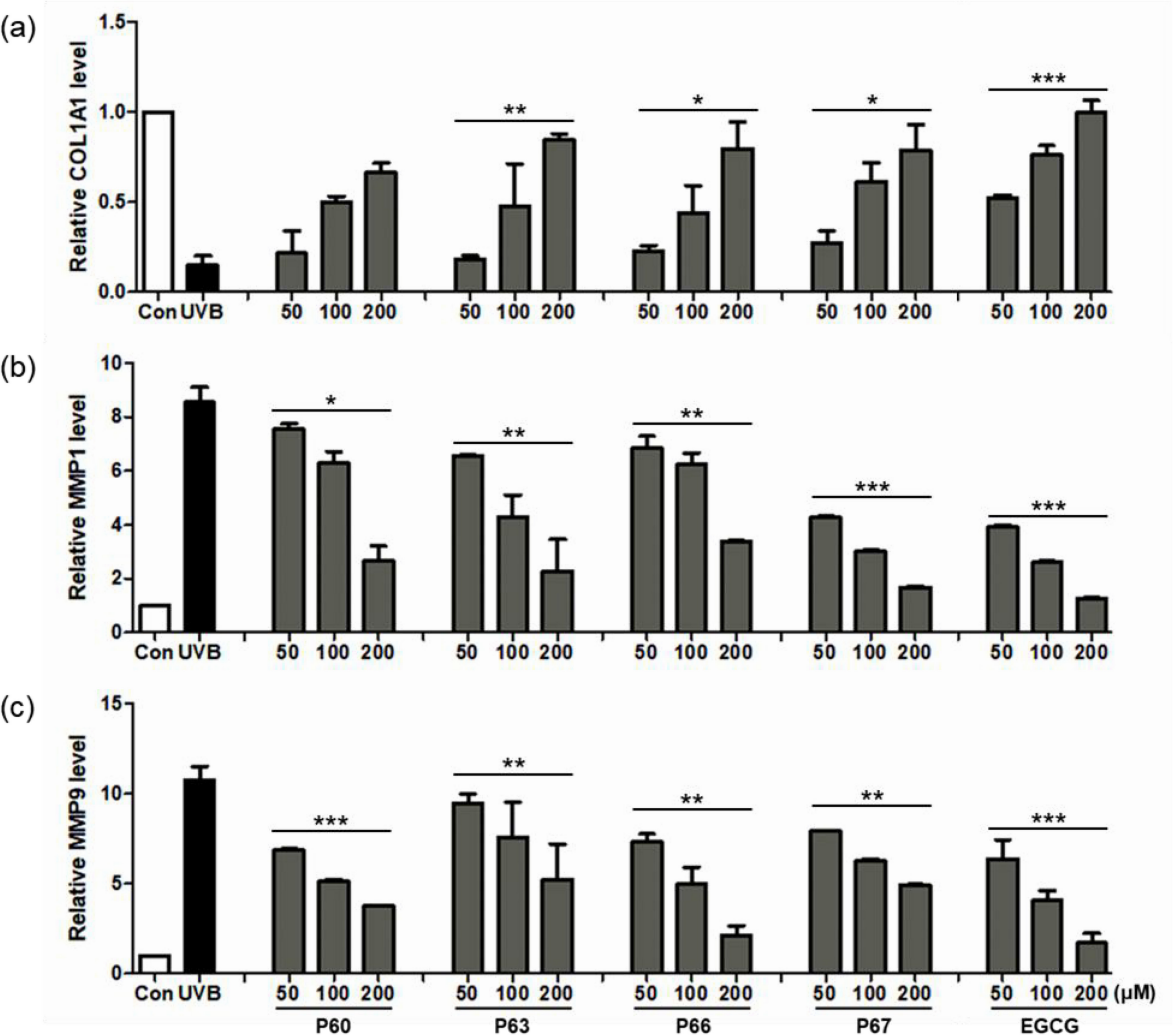
qPCR analysis of photoaging‐related transcripts. (a–c) Relative mRNA levels in ultraviolet B‐exposed CCD‐986sk fibroblasts treated with candidate peptides. COL1A1 increased (a), whereas MMP1 (b) and MMP9 (c) decreased compared with those after only UVB exposure. Results are expressed as fold change (2^−ΔΔCt^) normalized to β‐actin; mean ± SD (*n* = 3) with significance indicated (*p* < 0.05, *p* < 0.01, *p* < 0.001).

UVB exposure increased *MMP1* and *MMP9* mRNA levels and decreased *COL1A1* mRNA level in CCD‐986sk fibroblasts, confirming UVB‐induced photoaging (Figure 3). In contrast, treatment with four selected peptides (60, 63, 66, and 67) dose‐dependently reversed this effect. The expression of *COL1A1* transcripts was restored, whereas that of *MMP1*/*MMP9* decreased relative to that in the UVB group (Figure 5). Taken together, these qPCR data are consistent with the results of collagenase inhibition and PIP recovery, supporting the anti‐photoaging effect of simultaneous collagen synthesis stimulation and collagen degradation suppression.

### 3.6 Candidate Peptides Attenuate UVB‐Induced MMP1/MMP9 Secretion

**FIGURE 6:**
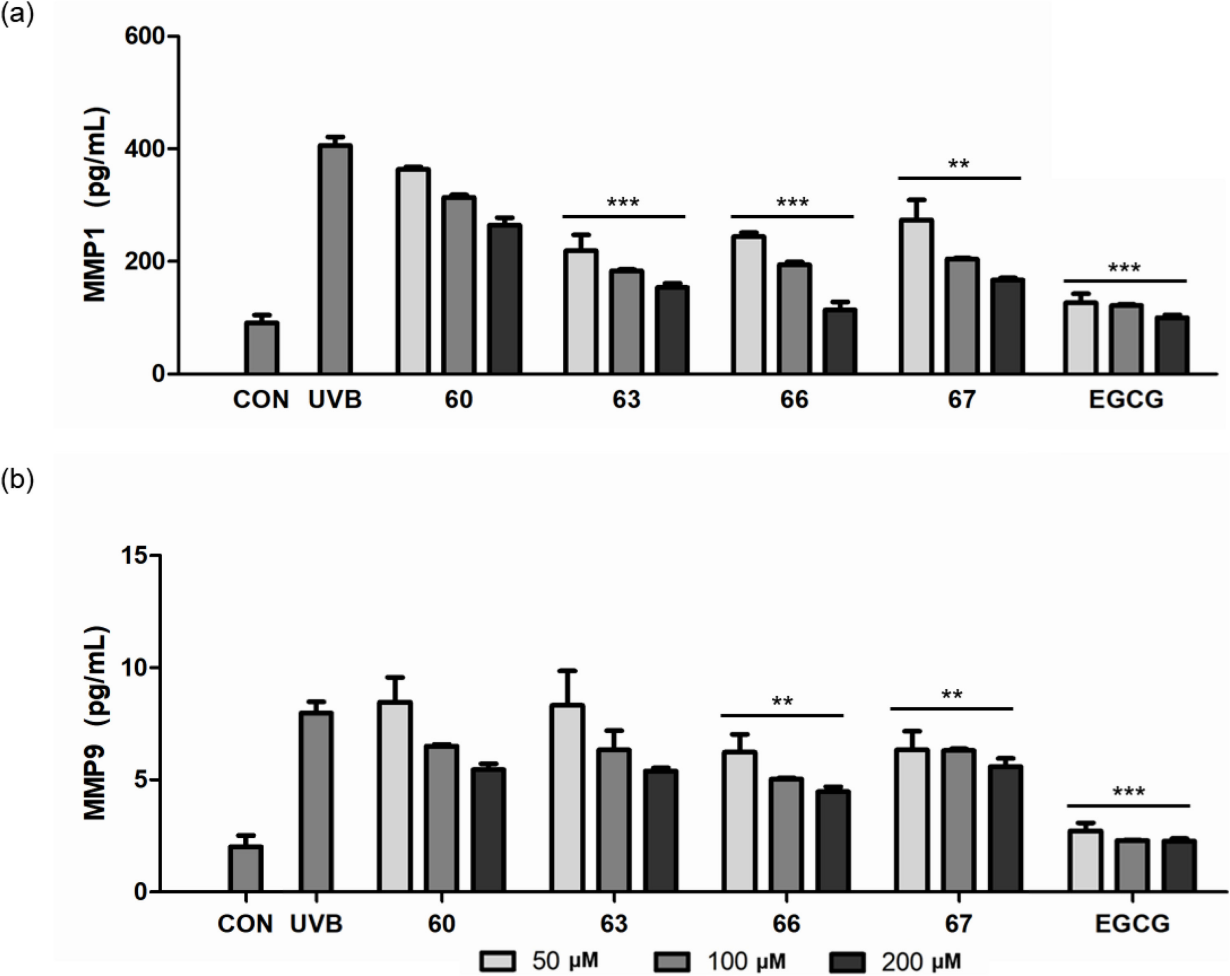
ELISA quantification of matrix metalloproteinase (MMP)‐1 and MMP‐9 secretion. (a, b) UVB elevated MMP‐1 (a) and MMP‐9 (b) protein levels in CCD‐986sk cultures, whereas treatment with candidate peptides reduced secretion in a dose‐dependent manner; P66 and P84 showed pronounced suppression at 200 μM, approaching EGCG. Data are presented as mean ± SD (*n* = 3).

ELISA was performed to examine MMP1 and MMP9 expression. As expected, UVB irradiation markedly increased the secretion of both metalloproteases relative to that in the non‐irradiated controls (Figure 6). In contrast, treatment with the four candidate peptides (60, 63, 66, and 67) concentration‐dependently decreased both MMP1 and MMP9 levels. Notably, 200 μM peptide 66 significantly reduced MMP1 and MMP9 levels than UVB irradiation. Overall, peptide treatment showed inhibitory effects similar to those of the positive control, EGCG. These results support the efficacy of these candidate peptides in mitigating UVB‐induced photoaging.

## 4 Discussion

This study establishes a novel workflow that connects the stress responses of γ‐irradiated *D. radiodurans* and *C. neoformans* to the identification of short peptides with antioxidant properties. Transcriptome profiling revealed a consistent enhancement of stress response programs, including DNA repair, protein repair through proteasome pathways, and the maintenance of ion and energy homeostasis. These responses are consistent with reduced radiation‐induced oxidative damage. Based on this biological transcriptomic signature, we streamlined an extensive peptide library using biochemical property filters and seven ML algorithms. Because the probability score distributions differed for each classifier, model‐specific thresholds were applied to increase the specificity of the selective screening. AdaBoost showed a relatively compressed score distribution; therefore, a lower threshold (0.53) was used to enable balanced participation within the majority voting ensemble. The inclusion of AdaBoost also contributed to improving the robustness of the consensus‐based candidate selection by increasing the diversity of classifiers. We then employed structure‐based docking to the KEAP1–NRF2 recognition pocket, which helped prioritize the final candidates with antioxidant activity. From this selection, the top 21 peptides were synthesized, and of these, four peptides, 60, 63, 66, and 67, showed convergent anti‐photoaging activity in vitro.

The two‐step screening process, which included collagenase inhibition and PIP recovery, assessed both the indirect and direct effects of peptides on wrinkle formation. Several candidate peptides attenuated type I procollagen levels in UVB‐exposed fibroblasts and decreased collagenase activity. These consistent results support the protective effects of candidate peptides on the ECM and allow for their further validation. The anti‐photoaging efficacy of the KEAP1 docking peptide reinforces the idea that short peptides derived from γ‐irradiated microbial proteomes can influence antioxidant signaling pathways and mitigate the cascade damage caused by oxidative stress. Although these peptides demonstrated lower potency than the positive control EGCG in vitro, their safety, processing ease, and targetability profiles make them promising candidates for optimization and development as anti‐photoaging agents.

This study had two important limitations. First, the experimental validation was limited. Although peptides with antioxidant activity were identified using KEAP1 molecular docking, this study focused solely on their anti‐photoaging effects in UVB‐exposed fibroblasts. However, oxidative stress can lead to various damages, including inflammation, aging, tissue injury, and pigmentation, none of which were addressed in this study [19]. Therefore, further studies should evaluate the efficacy of these peptides in various oxidative stress‐associated diseases by measuring standard antioxidant endpoints, anti‐inflammatory markers, wound‐healing indicators, and melanogenesis. Additionally, in vivo photoaging models are needed to improve confidence in clinical translation, and confirmation of the nuclear translocation of NRF2 is required to provide direct evidence for KEAP1–NRF2 complex disruption.

## 5 Conclusion

This study presents a multipronged strategy that combines omics, ML, and molecular docking to facilitate the systematic discovery and prioritization of antioxidant peptides. This integration could be applied to not only KEAP1 but also other oxidative stress pathways and targets, thereby expanding the range of mechanistic applications and providing a platform for the rational optimization of lead candidates. Based on the currently identified peptides (60, 63, 66, and 67), pharmacochemical optimization can enhance their stability, permeability, and efficacy. Further studies on formulation and targeted delivery related to dermal application, along with testing for synergy with small‐molecule antioxidants, will help clarify its clinical potential. Collectively, the pipeline outlined in this study offers a scalable approach for discovering functional peptides derived from stress‐adapted proteomes. This study aimed to identify novel anti‐photoaging lead compounds that preserve the ECM and activate the NRF2 pathway.

## Author Contributions

**Hanui Lee (H.L.):** conceptualization, methodology, formal analysis, data curation, investigation, visualization, writing – original draft. **Gyeong Han Jeong (G.H.J.):** data curation, formal analysis, software, visualization. **Ji Wan Choi (J.W.C.):** investigation, validation, resources, writing – review and editing. **Taehwan Kim (T.K.):** software, formal analysis, validation. **Younhee Shin (Y.S.):** software, formal analysis, validation. **Jaewon Lim (J.L.):** software, data curation, formal analysis. **Kwang‐Woo Jung (K.W.J.):** methodology, software, validation. **Byung Yeoup Chung (B.Y.C.):** resources, funding acquisition, project administration. **Seung Sik Lee (S.S.L.):** conceptualization, supervision, funding acquisition, project administration, writing – review and editing.

## Funding

This research was supported by the KAERI Institutional Program (Project No. 523310‐26) grant funded by the Nuclear R&D Program of the Ministry of Science and ICT.

## Ethics Statement

The authors have nothing to report.

## Consent

The authors have nothing to report.

## Conflicts of Interest

The authors declare no conflicts of interest.

## Supporting information


**Figure S1:** HPLC chromatogram of peptide 60.
**Figure S2:** MS spectrum of peptide 60.
**Figure S3:** HPLC chromatogram of peptide 63.
**Figure S4:** MS spectrum of peptide 63.
**Figure S5:** HPLC chromatogram of peptide 66.
**Figure S6:** MS spectrum of peptide 66.
**Figure S7:** HPLC chromatogram of peptide 67.
**Figure S8:** MS spectrum of peptide 67.
**Figure S9:** Effects of candidate peptides on the viability of CCD986‐sk fibroblasts.
**Table S1:** Average docking scores of antioxidant candidate peptides derived from *C. neoformans* (average score ≤ −8.07).
**Table S2:** Average docking scores of antioxidant candidate peptides derived from *D. radiodurans* (average score ≤ −8.07).

## Data Availability

The data underlying this article are available in the article and online Supporting Information.

## References

[1] J. Wenk , P. Brenneisen , C. Meewes , et al., “UV‐Induced Oxidative Stress and Photoaging,” Current Problems in Dermatology 29 (2001): 83–94, 10.1159/000060656. 11225204

[2] M. Wlaschek , I. Tantcheva‐Poór , L. Naderi , et al., “Solar UV Irradiation and Dermal Photoaging,” Journal of Photochemistry and Photobiology B: Biology 63 (2001): 41–51, 10.1016/S1011-1344(01)00201-9. 11684450

[3] P. Pittayapruek , J. Meephansan , O. Prapapan , M. Komine , and M. Ohtsuki , “Role of Matrix Metalloproteinases in Photoaging and Photocarcinogenesis,” International Journal of Molecular Sciences 17 (2016): 868, 10.3390/ijms17060868. 27271600 PMC4926402

[4] T. Quan , Z. Qin , W. Xia , Y. Shao , J. J. Voorhees , and G. J. Fisher , “Matrix‐Degrading Metalloproteinases in Photoaging,” Journal of Investigative Dermatology. Symposium Proceedings 14 (2009): 20–24, 10.1038/jidsymp.2009.8. 19675548 PMC2909639

[5] S. B. Fonseca , M. P. Pereira , and S. O. Kelley , “Recent Advances in the Use of Cell‐Penetrating Peptides for Medical and Biological Applications,” Advanced Drug Delivery Reviews 61 (2009): 953–964, 10.1016/j.addr.2009.06.001. 19538995

[6] J. J. Nestor , “The Medicinal Chemistry of Peptides,” Current Medicinal Chemistry 16 (2009): 4399–4418, 10.2174/092986709789712907. 19835565

[7] W. Liu , H. Tang , L. Li , X. Wang , Z. Yu , and J. Li , “Peptide‐Based Therapeutic Cancer Vaccine: Current Trends in Clinical Application,” Cell Proliferation 54 (2021): e13025, 10.1111/cpr.13025. 33754407 PMC8088465

[8] Y. A. Haggag , A. A. Donia , M. A. Osman , and S. A. El‐Gizawy , “Peptides as Drug Candidates: Limitations and Recent Development Perspectives,” Biomedical Journal of Scientific & Technical Research 8 (2018): 6221–6226, 10.26717/BJSTR.2018.08.001694.

[9] C. Yu and J. H. Xiao , “The Keap1‐Nrf2 System: A Mediator Between Oxidative Stress and Aging,” Oxidative Medicine and Cellular Longevity 2021 (2021): 6635460, 10.1155/2021/6635460. 34012501 PMC8106771

[10] M. Kobayashi and M. Yamamoto , “Molecular Mechanisms Activating the Nrf2‐Keap1 Pathway of Antioxidant Gene Regulation,” Antioxidants & Redox Signaling 7 (2005): 385–394, 10.1089/ars.2005.7.385. 15706085

[11] D. Slade and M. Radman , “Oxidative Stress Resistance in *Deinococcus radiodurans* ,” Microbiology and Molecular Biology Reviews 75 (2011): 133–191, 10.1128/MMBR.00015-10. 21372322 PMC3063356

[12] S. M. Brown and J. K. Lodge , “ *Cryptococcus neoformans*, a Fungus Under Stress,” Current Opinion in Microbiology 10 (2007): 320–325, 10.1016/j.mib.2007.05.014. 17707685 PMC2570326

[13] K. J. Kwon‐Chung , J. A. Fraser , T. L. Doering , et al., “ *Cryptococcus neoformans* and *Cryptococcus gattii*, the Etiologic Agents of Cryptococcosis,” Cold Spring Harbor Perspectives in Medicine 4 (2014): a019760, 10.1101/cshperspect.a019760. 24985132 PMC4066639

[14] M. M. Cox and J. R. Battista , “ *Deinococcus radiodurans*—The Consummate Survivor,” Nature Reviews. Microbiology 3 (2005): 882–892, 10.1038/nrmicro1264. 16261171

[15] D. C. Rio , M. Ares , G. J. Hannon , and T. W. Nilsen , “Purification of RNA Using Trizol (TRI Reagent),” Cold Spring Harbor Protocols 2010 (2010): pdb.prot5439, 10.1101/pdb.prot5439. 20516177

[16] P. Kumar , A. Nagarajan , and P. D. Uchil , “Analysis of Cell Viability by the MTT Assay,” Cold Spring Harbor Protocols 2018 (2018): pdb.prot095505, 10.1101/pdb.prot095505. 29858338

[17] P. P. Shinde and S. Shah , “A Review of Machine Learning and Deep Learning Applications,” in 2018 Fourth International Conference on Computing Communication Control and Automation (ICCUBEA) (IEEE, 2018), 1–6, 10.1109/ICCUBEA.2018.8697857.

[18] A. Lau , S. A. Whitman , M. C. Jaramillo , and D. D. Zhang , “Arsenic‐Mediated Activation of the Nrf2‐Keap1 Antioxidant Pathway,” Journal of Biochemical and Molecular Toxicology 27 (2013): 99–105, 10.1002/jbt.21463. 23188707 PMC3725327

[19] S. Chatterjee , “Oxidative Stress, Inflammation, and Disease,” in Oxidative Stress and Biomaterials (Elsevier, 2016), 35–58, 10.1016/B978-0-12-803269-5.00002-1.

