# Supplementary material for: Integrative Identification of Anti‐Photoaging Peptides From Stress‐Tolerant Microorganisms via Machine Learning and KEAP1–NRF2 Docking
Source: J Pept Sci. 2026 Jul 1;32(8):e70115. doi: 10.1002/psc.70115 (PMC13323872; doi:10.1002/psc.70115)
Supplement: Supplementary file 1 — Figure S1: HPLC chromatogram of peptide 60. Figure S2: MS spectrum of peptide 60. Figure S3: HPLC chromatogram of peptide 63. Figure S4: MS spectrum of peptide 63. Figure S5: HPLC chromatogram of peptide 66. Figure S6: MS spectrum of peptide 66. Figure S7: HPLC chromatogram of peptide 67. Figure S8: MS spectrum of peptide 67. Figure S9: Effects of candidate peptides on the viability of CCD986‐sk fibroblasts. Table S1: Average docking scores of antioxidant candidate peptides derived from C. neoformans (average score ≤ −8.07). Table S2: Average docking scores of antioxidant candidate peptides derived from D. radiodurans (average score ≤ −8.07). [file PSC-32-e70115-s001.docx]

**Supplementary material**

**Integrative identification of anti-photoaging peptides from stress-tolerant microorganisms via machine learning and KEAP1–NRF2 docking**

Hanui Lee ^1^, Gyeong Han Jeong ^1^, Ji Wan Choi ^1,2^, Taehwan Kim ^1^, Byung Yeoup Chung ^1^, Seung Sik Lee ^1,2,*^

^1^ Research Division for Biotechnology, Advanced Radiation Technology Institute (ARTI), Korea Atomic Energy Research Institute (KAERI), Jeongeup 56212, Republic of Korea

^2^ Department of Radiation Science, University of Science and Technology (UST), Daejeon 34113, Republic of Korea

* Corresponding Author.

*E-mail address*: sslee@kaeri.re.kr (S.S. Lee)

**Contents**

**Figure S1.** HPLC chromatogram of peptide 60.

**Figure S2.** MS spectrum of peptide 60.

**Figure S3.** HPLC chromatogram of peptide 63.

**Figure S4.** MS spectrum of peptide 63.

**Figure S5.** HPLC chromatogram of peptide 66.

**Figure S6.** MS spectrum of peptide 66.

**Figure S7.** HPLC chromatogram of peptide 67.

**Figure S8.** MS spectrum of peptide 67.

**Figure S9.** Effects of candidate peptides on the viability of CCD986-sk fibroblasts.

**Table S1.** Average docking scores of antioxidant candidate peptides derived from C. neoformans (average score ≤ −8.07).

**Table S2.** Average docking scores of antioxidant candidate peptides derived from D. radiodurans (average score ≤ −8.07).

**
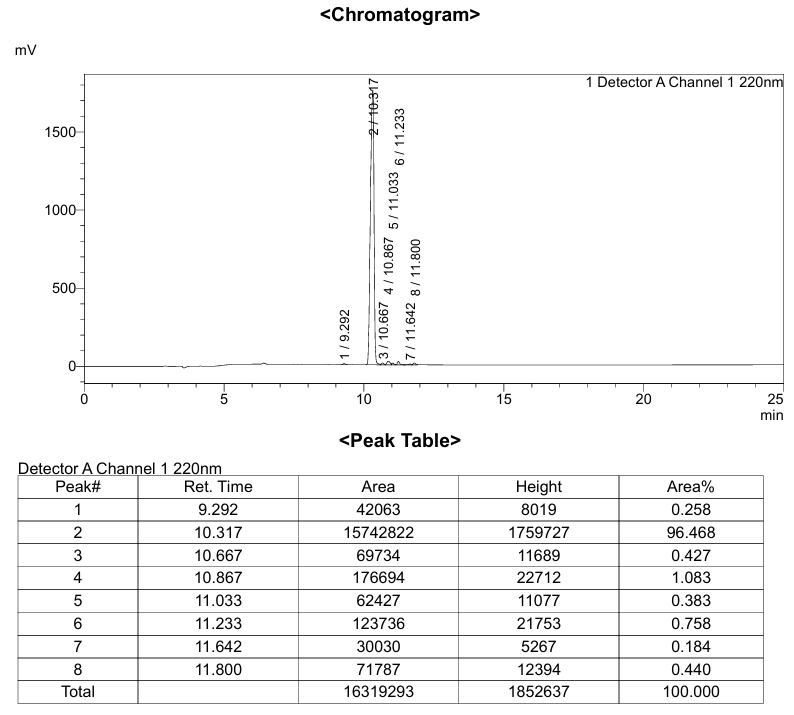
**

**Figure S1.** HPLC chromatogram of peptide 60.


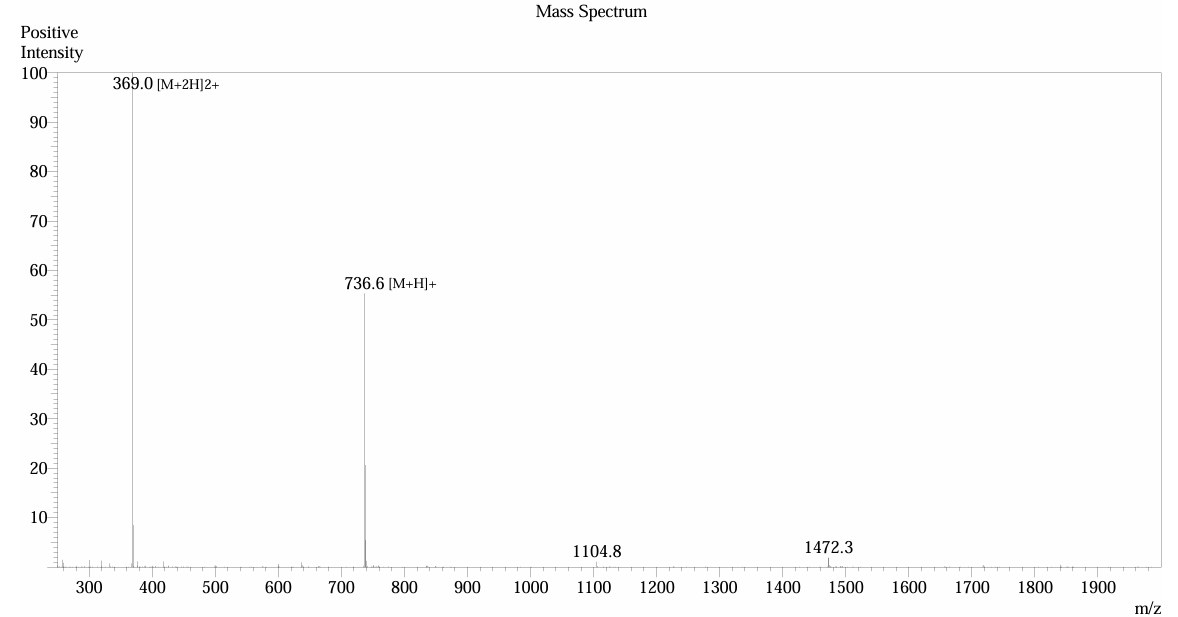


**Figure S2.** MS spectrum of peptide 60.


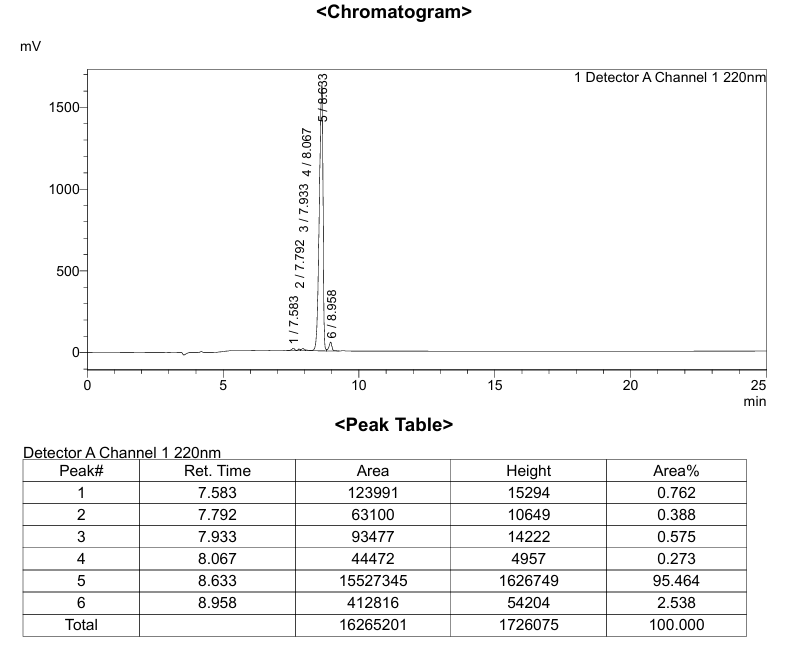


**Figure S3.** HPLC chromatogram of peptide 63.


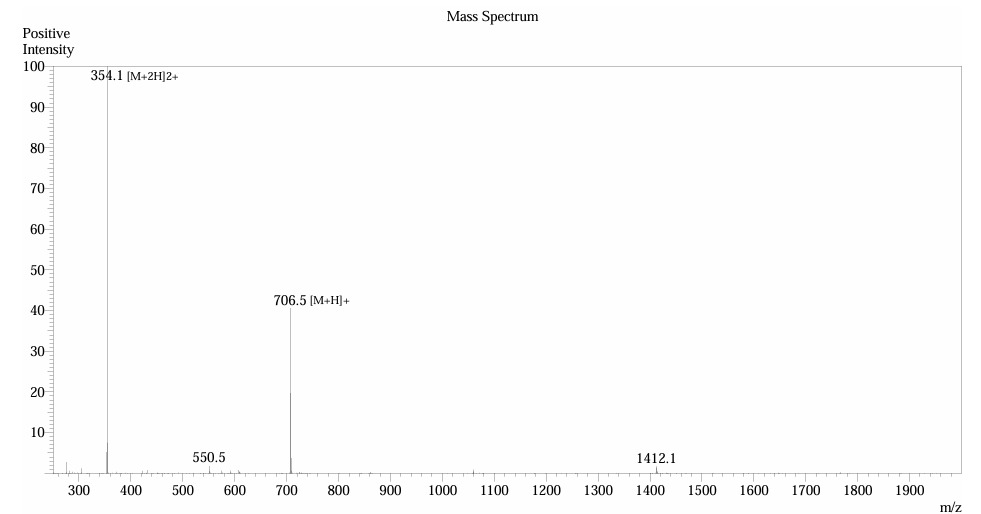


**Figure S4.** MS spectrum of peptide 63.


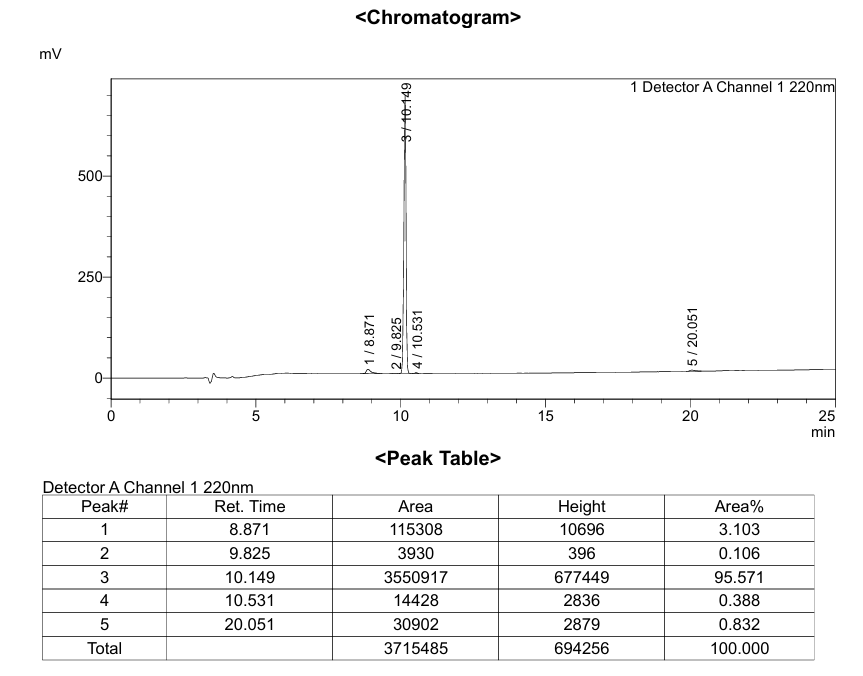


**Figure S5.** HPLC chromatogram of peptide 66.


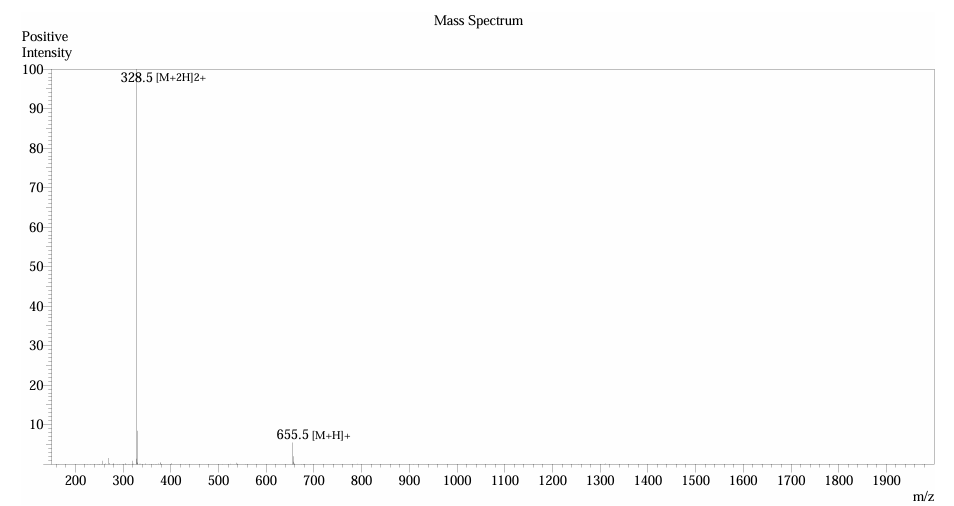


**Figure S6.** MS spectrum of peptide 66.


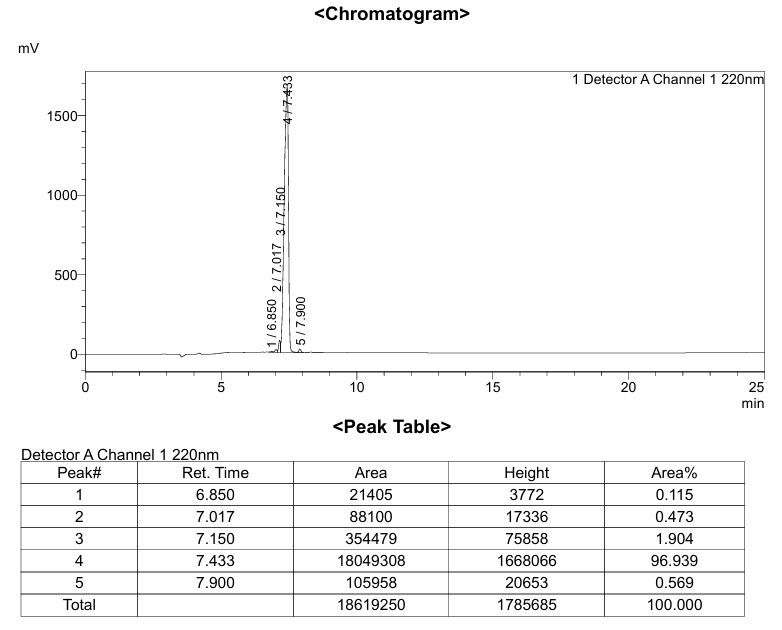


**Figure S7.** HPLC chromatogram of peptide 67.


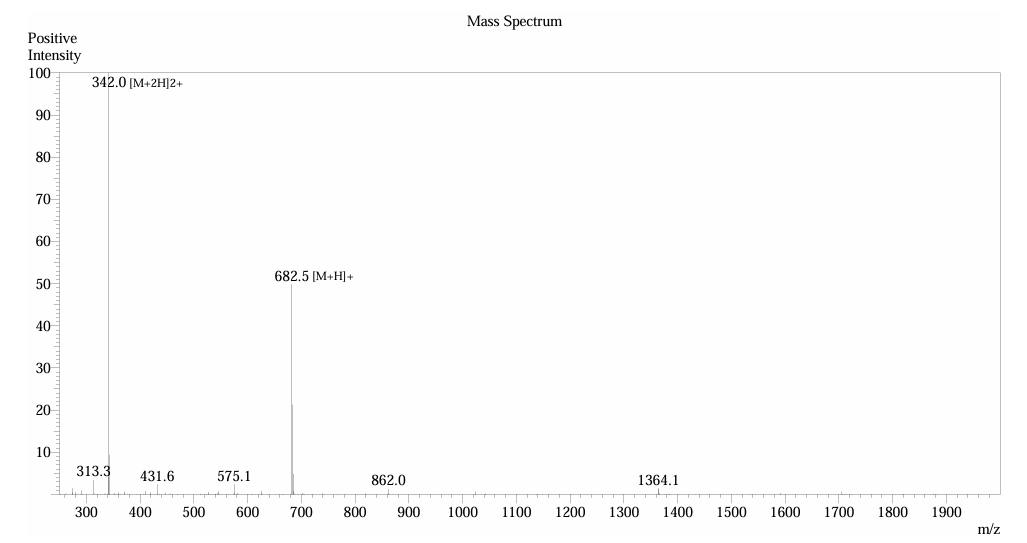


**Figure S8.** MS spectrum of peptide 67.

**
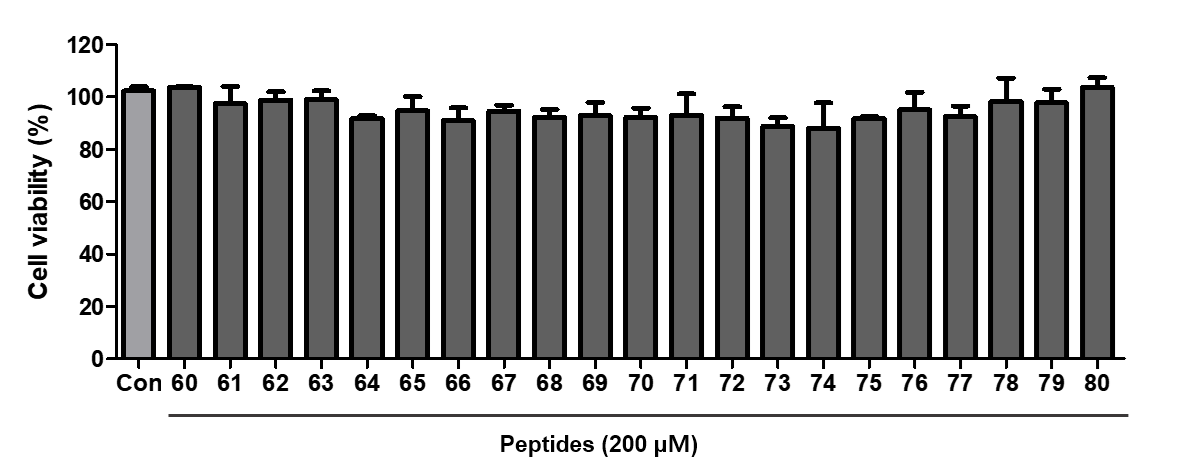
**

**Figure S9.** Effects of candidate peptides on the viability of CCD986-sk fibroblasts.

| **Table S1** Average docking scores of antioxidant candidate peptides derived from *C. neoformans* (average score ≤ −8.07). | | | |
| --- | --- | --- | --- |
| Peptide | Sequences | Length | Average Score |
| XP_012046146.1 TPR repeat-containing protein [Cryptococcus neoformans var. grubii H99]_23_Arg-C_360_6_735.92788_9.73\|RPG | VHVLIR | 6 | -12.6996 |
| XP_012050098.1 large subunit ribosomal protein L2 [Cryptococcus neoformans var. grubii H99]_76_Pepsin-pH=2_79_6_753.90238_9.73\|RPG | RHVVFP | 6 | -11.7457 |
| XP_012048691.1 GTPase [Cryptococcus neoformans var. grubii H99]_1_Arg-C_8_6_705.85818_9.73\|RPG | HIAPIR | 6 | -10.6383 |
| XP_012047669.1 hypothetical protein CNAG_04086 [Cryptococcus neoformans var. grubii H99]_103_Chymotrypsin-low_213_6_733.91198_9.73\|RPG | IVLRPH | 6 | -10.2389 |
| XP_012050939.1 alpha-ketoglutarate catabolism dioxygenase [Cryptococcus neoformans var. grubii H99]_3_Proline-endopeptidase_302_6_705.85838_9.73\|RPG | VVRVHP | 6 | -10.1952 |
| XP_012051711.1 Fe-S protein assembly co-chaperone HscB [Cryptococcus neoformans var. grubii H99]_0_Proline-endopeptidase_7_7_851.07778_9.73\|RPG | MVLRVHP | 7 | -9.99446 |
| XP_012053362.1 cytoplasmic protein [Cryptococcus neoformans var. grubii H99]_59_Arg-C_1038_6_681.79288_9.73\|RPG | GTVHIR | 6 | -9.94393 |
| XP_012053177.1 hypothetical protein CNAG_06162 [Cryptococcus neoformans var. grubii H99]_352_Proteinase-K_185_5_654.82848_9.73\|RPG | MRIHV | 5 | -9.93443 |
| XP_012051283.1 hypothetical protein CNAG_03475 [Cryptococcus neoformans var. grubii H99]_42_Arg-C_406_6_681.79288_9.73\|RPG | HVITGR | 6 | -9.77697 |
| XP_012050865.1 nuclear protein [Cryptococcus neoformans var. grubii H99]_98_Chymotrypsin-low_500_5_622.76848_9.73\|RPG | VIVRH | 5 | -9.68159 |
| XP_012047802.1 hypothetical protein CNAG_03022 [Cryptococcus neoformans var. grubii H99]_56_Neutrophil-elastase_120_6_725.90728_9.73\|RPG | HIMVRA | 6 | -9.388 |
| XP_012051316.1 chromosome transmission fidelity protein 8 [Cryptococcus neoformans var. grubii H99]_59_Thermolysin_6_6_765.97198_9.73\|RPG | MRIHLP | 6 | -9.21778 |
| XP_012052776.1 hypothetical protein CNAG_01814 [Cryptococcus neoformans var. grubii H99]_5_ProAlanase_17_6_748.88318_9.73\|RPG | NRHILP | 6 | -9.019 |
| XP_012051487.1 NADPH2 dehydrogenase [Cryptococcus neoformans var. grubii H99]_3_Bromelain_27_6_725.90728_9.73\|RPG | HRIVMA | 6 | -8.95046 |
| XP_012049872.1 nuclear export factor [Cryptococcus neoformans var. grubii H99]_812_Elastase_206_5_723.87588_9.73\|RPG | RWHIL | 5 | -8.91933 |
| XP_012048117.1 ATP-binding cassette, subfamily D (ALD), peroxisomal long-chain fatty acid import protein [Cryptococcus neoformans var. grubii H99]_386_Elastase_90_5_684.83928_9.73\|RPG | FHIRL | 5 | -8.87027 |
| XP_012047221.1 hypothetical protein CNAG_03783 [Cryptococcus neoformans var. grubii H99]_118_Chymotrypsin-low_248_5_636.79528_9.73\|RPG | VHIRL | 5 | -8.83519 |
| XP_012048961.1 hypothetical protein CNAG_01357 [Cryptococcus neoformans var. grubii H99]_261_Proteinase-K_197_5_580.68778_9.73\|RPG | GHIRV | 5 | -8.82486 |
| XP_012047756.1 membrane protein [Cryptococcus neoformans var. grubii H99]_403_Elastase_329_6_798.94318_9.73\|RPG | RVFQHL | 6 | -8.70426 |
| XP_012046834.1 translation initiation factor 3 subunit I [Cryptococcus neoformans var. grubii H99]_28_Chymotrypsin-high_321_5_695.82228_9.73\|RPG | VRVHW | 5 | -8.6593 |
| XP_012047700.1 dethiobiotin synthase [Cryptococcus neoformans var. grubii H99]_248_Thermolysin_12_5_685.78378_9.73\|RPG | FRVHQ | 5 | -8.64455 |
| XP_012048330.1 preconditioning-inducible protein [Cryptococcus neoformans var. grubii H99]_35_ProAlanase_195_6_719.88518_9.73\|RPG | VRIVHP | 6 | -8.57545 |
| XP_012047388.1 hypothetical protein CNAG_03685 [Cryptococcus neoformans var. grubii H99]_166_Chymotrypsin-low_338_5_636.79528_9.73\|RPG | LIVRH | 5 | -8.55708 |
| XP_012047427.1 large subunit ribosomal protein L10-like [Cryptococcus neoformans var. grubii H99]_84_Thermolysin_93_5_606.72578_9.73\|RPG | VRVHP | 5 | -8.54288 |
| XP_012048318.1 hypothetical protein CNAG_03076 [Cryptococcus neoformans var. grubii H99]_20_ProAlanase_94_5_634.77938_9.73\|RPG | HIIRP | 5 | -8.53819 |
| XP_012052703.1 sulfonate dioxygenase [Cryptococcus neoformans var. grubii H99]_91_Neutrophil-elastase_264_6_719.88518_9.73\|RPG | HPIVRV | 6 | -8.39688 |
| XP_012051513.1 transcription initiation factor TFIIH subunit 2 [Cryptococcus neoformans var. grubii H99]_319_Proteinase-K_240_5_582.66028_9.73\|RPG | GHIRT | 5 | -8.38974 |
| XP_012048084.1 T-complex protein 1 subunit gamma [Cryptococcus neoformans var. grubii H99]_355_Proteinase-K_122_6_757.89368_9.73\|RPG | RHIHPV | 6 | -8.3794 |
| XP_012046146.1 TPR repeat-containing protein [Cryptococcus neoformans var. grubii H99]_249_Chymotrypsin-low_358_5_622.76848_9.73\|RPG | RVHVL | 5 | -8.37767 |
| XP_012051192.1 minichromosome maintenance protein 2 [Cryptococcus neoformans var. grubii H99]_131_Pepsin-pH1.3_788_6_721.85768_9.73\|RPG | PITVRH | 6 | -8.36783 |
| XP_012052404.1 peptide-N4-(N-acetyl-beta-glucosaminyl)asparagine amidase [Cryptococcus neoformans var. grubii H99]_128_Neutrophil-elastase_263_7_946.12278_9.73\|RPG | LRHWVHV | 7 | -8.19703 |
| … | … | … | … |

| **Table S2** Average docking scores of antioxidant candidate peptides derived from *D. radiodurans* (average score ≤ −8.07). | | | |
| --- | --- | --- | --- |
| Peptide | Sequences | Length | Average Score |
| WP_027479933.1 membrane protein insertase YidC [Deinococcus radiodurans]_132_ProAlanase_243_7_804.99078_9.73\|RPG | HALIVRP | 7 | -10.2892 |
| WP_010888963.1 hypothetical protein [Deinococcus radiodurans]_10_Bromelain_65_7_734.85648_9.73\|RPG | VRVPGHA | 7 | -10.1488 |
| WP_010888358.1 cytochrome P450 [Deinococcus radiodurans]_391_Proteinase-K_311_5_620.75258_9.73\|RPG | RVHPI | 5 | -9.84584 |
| WP_034351338.1 KAP family P-loop domain protein [Deinococcus radiodurans]_31_Arg-C_496_7_748.88328_9.73\|RPG | PAIHVGR | 7 | -9.68699 |
| WP_027480300.1 metalloenzyme domain protein [Deinococcus radiodurans]_188_Proteinase-K_95_7_748.88318_9.73\|RPG | HGLPGRL | 7 | -9.48204 |
| WP_010887551.1 DNA gyrase subunit B [Deinococcus radiodurans]_255_Thermolysin_152_5_674.80378_9.73\|RPG | LHHIR | 5 | -9.1704 |
| WP_010887363.1 aspartate 1-decarboxylase [Deinococcus radiodurans]_27_ProAlanase_81_6_723.89138_9.73\|RPG | AHLMRP | 6 | -9.15204 |
| WP_028328044.1 S8 family serine peptidase [Deinococcus radiodurans]_195_Thermolysin_94_6_694.79158_9.73\|RPG | LNVGRH | 6 | -9.12821 |
| WP_010889053.1 bifunctional nicotinamide-nucleotide adenylyltransferase/Nudix hydroxylase [Deinococcus radiodurans]_8_Arg-C_95_5_656.78568_9.73\|RPG | FVHVR | 5 | -9.05072 |
| WP_010889334.1 DDE-type integrase/transposase/recombinase [Deinococcus radiodurans]_86_Chymotrypsin-low_427_7_794.90898_9.73\|RPG | SLPRVSH | 7 | -8.8468 |
| WP_010887067.1 trans-aconitate 2-methyltransferase [Deinococcus radiodurans]_202_Proteinase-K_123_7_734.85638_9.73\|RPG | HLRPGGV | 7 | -8.54938 |
| WP_010886815.1 dihydroneopterin aldolase [Deinococcus radiodurans]_130_Proteinase-K_99_6_782.94388_9.93\|RPG | RVHKPF | 6 | -8.50653 |
| WP_034350675.1 branched-chain amino acid ABC transporter ATP-binding protein/permease [Deinococcus radiodurans]_19_Arg-C_329_7_826.95368_9.73\|RPG | AVTFPHR | 7 | -8.42859 |
| WP_010887486.1 PLP-dependent aminotransferase family protein [Deinococcus radiodurans]_327_Proteinase-K_118_6_725.84848_9.73\|RPG | RPGLHF | 6 | -8.40883 |
| WP_010887905.1 RNA-binding protein Rsr [Deinococcus radiodurans]_400_Elastase_313_5_634.77938_9.73\|RPG | RIHPL | 5 | -8.3926 |
| WP_010883957.1 two-component system response regulator RadR [Deinococcus radiodurans]_278_Thermolysin_289_5_620.75258_9.73\|RPG | VHLRP | 5 | -8.30715 |
| WP_231885354.1 sodium:proton antiporter [Deinococcus radiodurans]_369_Proteinase-K_150_6_707.83078_9.73\|RPG | HVPSRI | 6 | -8.07948 |
| WP_231885333.1 IS200/IS605 family element RNA-guided endonuclease TnpB [Deinococcus radiodurans]_84_Trypsin_101_6_702.85468_9.93\|RPG | KVGFPR | 6 | -7.98763 |
| WP_034350008.1 FAD:protein FMN transferase [Deinococcus radiodurans]_113_ProAlanase_153_7_826.95368_9.73\|RPG | TVHARFP | 7 | -7.8905 |
| WP_010889426.1 DUF4132 domain-containing protein [Deinococcus radiodurans]_1620_Proteinase-K_1324_5_634.77938_9.73\|RPG | RHPII | 5 | -7.74193 |
| WP_010887363.1 aspartate 1-decarboxylase [Deinococcus radiodurans]_10_ProAlanase_81_5_652.81258_9.73\|RPG | HLMRP | 5 | -7.67076 |
| WP_027479567.1 imidazoleglycerol-phosphate dehydratase HisB [Deinococcus radiodurans]_68_Pepsin-pH1.3_156_5_636.79528_9.73\|RPG | LHVRL | 5 | -7.55389 |
| WP_010887187.1 glycerophosphodiester phosphodiesterase [Deinococcus radiodurans]_179_Proteinase-K_115_5_580.68778_9.73\|RPG | RVHGL | 5 | -7.52717 |
| WP_034350644.1 LysR family transcriptional regulator [Deinococcus radiodurans]_8_ProAlanase_52_6_767.92918_9.73\|RPG | LFHRVP | 6 | -7.50125 |
| WP_010889334.1 DDE-type integrase/transposase/recombinase [Deinococcus radiodurans]_560_Proteinase-K_425_6_707.83078_9.73\|RPG | HSLPRV | 6 | -7.41776 |
| WP_027480205.1 Sectered polysaccharide deacetylase [Deinococcus radiodurans]_44_Pepsin-pH1.3_258_7_750.85588_9.73\|RPG | HSPRVVG | 7 | -7.39114 |
| WP_010888600.1 enoyl-ACP reductase [Deinococcus radiodurans]_260_Proteinase-K_205_6_755.87478_9.73\|RPG | RSIPHF | 6 | -7.35813 |
| WP_027479703.1 transcriptional repressor LexA [Deinococcus radiodurans]_25_Pepsin-pH1.3_163_5_715.85338_8.81\|RPG | KRLYH | 5 | -7.33658 |
| WP_010887551.1 DNA gyrase subunit B [Deinococcus radiodurans]_289_Thermolysin_265_6_699.82608_9.73\|RPG | VMRGTH | 6 | -7.31296 |
| WP_010888220.1 ATP-binding cassette domain-containing protein [Deinococcus radiodurans]_23_ProAlanase_181_5_634.77938_9.73\|RPG | LLHRP | 5 | -7.30231 |
| WP_010888007.1 pyridoxal 5'-phosphate synthase glutaminase subunit PdxT [Deinococcus radiodurans]_45_Pepsin-pH1.3_188_5_670.81248_9.73\|RPG | HRVFL | 5 | -7.29134 |
| … | … | … | … |
